# Descriptive Elements and Conceptual Structure of Glass Ceiling Research

**DOI:** 10.3390/ijerph18158011

**Published:** 2021-07-29

**Authors:** Salustiano Martínez-Fierro, María Paula Lechuga Sancho

**Affiliations:** INDESS (Research Universitary Institute for Sustainable Social Development), University of Cádiz, Campus de Jerez, 11406 Jerez de la Frontera, Cádiz, Spain; salustiano.martinez@uca.es

**Keywords:** glass ceiling, gender, bibliometric analysis, conceptual structure

## Abstract

Women make up more than half of the population of every society and are seen as the creators and instructors of the next generation. They are potentially significant human resources in the scientific, social, and cultural development of most countries and, consequently, special attention needs to be paid to the form of their occupational promotion. This paper describes the evolution of research on glass ceiling until July 2020. We compiled a database of 823 articles focused on the field and published in academic journals in the ISI WoS database. Bibliometric methods and techniques were used to describe the evolution of scientific activity, countries, and active institutions, most productive authors, most relevant sources, most influential documents, trend topics, and social structure researched. This determined the state of the art and described the evolution of the literature in this field, and it will help scholars refine existing and initiate new research agendas. A total of 846 documents were identified, and the results showed an upward trend in glass ceiling scientific production. Based on these analyses, possible forms of future research are proposed to advance toward the consolidation of this scientific discipline.

## 1. Introduction

Women’s social and economic position in the labor market, their preparation, and professional practice have changed over time, evolving favorably. However, women and men have different professional development and career training [1]. As the hierarchical level of the company increases, the presence of women decreases, while the presence of men increases, being in some cases total male presence [2]. This unequal presence of men and women in management positions is widely known and demonstrated by various research studies [3,4,5]. Several studies have tried to explain different aspects of these inequalities [1], identifying other metaphors [6], including what is known as the “glass ceiling” [7]. 

The metaphor of the “glass ceiling” has been widely used in the gender and business management literature [1]. The term “glass ceiling” was first used in the United States in the 1970s [8]. This metaphor represents the invisible barriers that hinder not only women but also other highly qualified minorities in their career progression and access to positions at the higher levels of the organization, primarily executive and managerial positions [8,9,10].

This concept reflects most situations of discrimination in the workplace [11] but has been generally used in the literature on working women [10]. Previous research confirms that many working women still have to work under a “glass ceiling” that they cannot break since women have less access to higher professional positions and income [10]. However, as highlighted in the literature, the presence of women in management positions is important to achieve gender balance as a symbolic act within these organizations and because this diversity can have numerous beneficial impacts on the organizations. This phenomenon can be explained both from the perspective of the agency theory and the upper echelon theory [12]. A gender-diversified board of directors causes companies to have a lower level of agency costs [13]. Women’s participation in management teams is associated with improvements in firm performance [14]. This performance is usually measured by financial results [15], corporate debt levels [16] and corporate social responsibility [17]. In this sense, Rodríguez-Fernández et al. [18] show that the financial performance of companies improves with the presence of women in the management team, as well as having a positive impact on company sales. Ruiz-Jiménez et al. [19] showed that gender diversity positively moderates the relationship between combining knowledge and innovation performance.

To advance this line of research and to be able to make an agenda of new practices and topics related to the glass ceiling, it is necessary to know and understand the progress of this topic. Specific research has been carried out on the above topics, but there are no other bibliometric studies that review the literature on the glass ceiling in a broad way. Therefore, we consider that our paper is essential to contribute to scientific knowledge by providing a global vision of this research topic. Bibliometric analysis can help understand the structure and development of this research topic and its evolution [20]. There are some bibliometric research studies on different metaphors of gender inequalities [6,21], but we found very few bibliometric studies on the glass ceiling [21,22,23,24]. More specifically, Carpenter et al. [24] conducted a bibliometric analysis to examine the academic trend of women in neurosurgery. In the same vein, Ngaage et al. [23] compared female academic plastic surgeons’ academic titles and departmental leadership with a similar group of men. Da Rocha Grangeiro et al. [21] present a bibliometric analysis of the different metaphors related to gender inequality in leadership positions. In addition, Da Rocha Grangeiro et al. [6] used a systematic literature review of 1269 papers to identify and systematically summarize the relevant research on metaphors used to explain gender inequalities in the organizational context.

To fill this gap in the literature and to show a more realistic picture of the published research on the glass ceiling, our paper aims to carry out an exhaustive review of the scientific production of articles on the glass ceiling using bibliometric analysis. Essentially, we tried to provide answers to the following questions [25]:

How has the literature evolved?

In which sources are these articles published?

How have these sources grown?

Which are the most influential documents?

Which are the most frequent words?

Who are the most productive authors?

What countries show a more significant concern for this type of research?

What is the conceptual structure of the discipline like?

Once we have achieved these objectives, we will be able to guide glass ceiling research by considering innovative and emerging topics and suggest new lines of research. This study contributes theoretically and empirically to our understanding of the publication behaviors of glass-ceiling researchers.

To achieve our objectives, this research proceeds as follows. First, we describe the methodology, the origin of the data, and the analytical procedure. Second, we show the results of the descriptive bibliometric analysis and the conceptual structure. Finally, we present our conclusions and discuss the main implications, limitations, and future lines of research.

## 2. Materials and Methods

Bibliometric analysis constitutes a methodological innovation to the traditional literature review [26]. At the same time, it provides valuable information for researchers seeking to assess scientific activity [27]. A bibliometric analysis consists of applying statistical methods to determine quantitative and qualitative changes in a particular research topic, establishing the profile of publications in the topic, and detecting trends within a discipline [26]. More specifically, citation and co-citation analyses are based on purely quantitative approaches and are supported by the premise that citations are a reliable and valid indicator of scientific interactions between researchers and research institutions [28,29]. 

The research data used in this paper were obtained from the Web of Science (WoS) database, which includes scientific papers from all disciplines, in the top-ranked journals for the scientific community. Previous researchers have considered the WoS database as one of the most reliable sources of data for conducting systematic literature review studies [20,30,31].

Data were processed with Biblioshiny for Bibliometrix software [32], which is widely accepted as one of the most useful and comprehensive tools for this type of analysis [33]. 

The search query was performed in the WoS main collection, locating papers with the terms glass ceiling or roof ceiling in the title, abstract, or keyword fields. For efficient analysis, we limited the search to English-language articles only. Using a comprehensive language to provide an efficient bibliometric analysis provides us with several tools that compare keywords, article sources, and affiliations [34]. The query conducted yielded a total of 843 published papers on the glass ceiling to date. Book chapters and proceedings papers were removed as well as papers from 2021 so that the last year of analysis is complete, making the final sample a total of 823 research articles.

The correct application of the bibliometric methodology to the set of papers that make up the research conducted on a particular topic requires the examination of the main variables related to bibliometric research. Basing on some proposals from the academic literature [25], the selection of the main indices to be analyzed in this bibliometric study revolves around the following points: (i) trend in scientific production (years of publications and average of citations per year), (ii) title of the journals in which the papers have been published (core journals, source dynamics), (iii) documents (most cited papers, most frequent words, word cloud), (iv) most productive authors and affiliations, and (v) conceptual structure.

## 3. Results and Discussion

The descriptive characteristics of our sample is shown in Table 1. We found 823 papers published in 580 journals from 1987 to 2020. All articles used 1377 plus keywords (ID) and 1622 author’s keywords (DE). A total of 1764 authors wrote these papers, of which only 308 articles were written by a single author. Collaborative authorship is predominant in this research topic, as shown by the Collaboration Index, 2.85, or the ratio of papers per author (0.467).

### 3.1. Trend in Scientific Production

**Figure 1:**
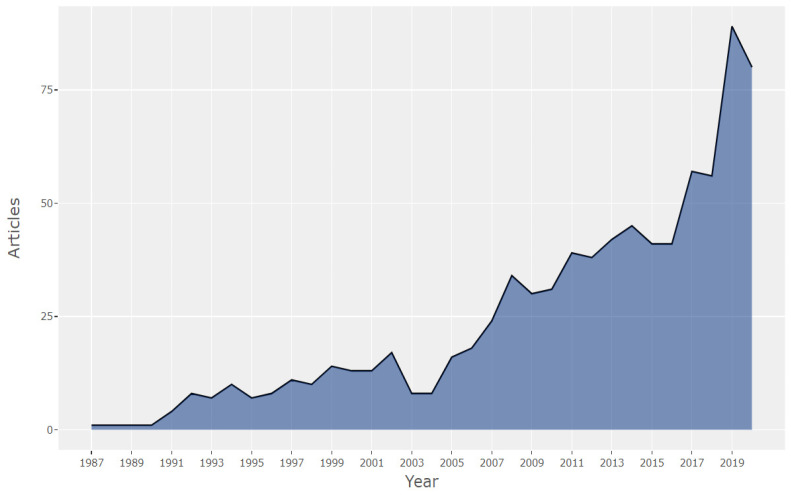
Annual scientific production.

In the first stage, the publications on the “glass ceiling” were analyzed by year. The evolution of scientific production during the period analyzed is shown in Figure 1. The general trend has been upward from 1990 to the present, increasing in the last few years. Publications in the first four years of the period analyzed are very scarce: one article per year. From 1991 onwards, two different periods can be observed. The first runs from 1992 to 2006; there is a slight increase in the number of papers, with an annual average of 11 papers. In 2007–2020, the rate of growth of publications on the glass ceiling is much higher, reaching almost 90 articles published in 2019. The most significant increase in publications occurs from 2016 onwards.

The data show how interest in this research topic has increased in recent years. The glass ceiling phenomenon is a hot topic for society and researchers in the social sciences. As a hot topic, the scientific community needs to look further into the discipline. In addition, a number of hitherto unexplored gaps such as the barriers that various minorities, not only women, face in gaining access to managerial positions in an organization should be further explored. This growing trend parallels the results obtained by Da Rocha Grangeiro et al. [6]. They state that there has been an increase in the interest of academics in studying gender inequality in the workplace in the last decade.

**Figure 2:**
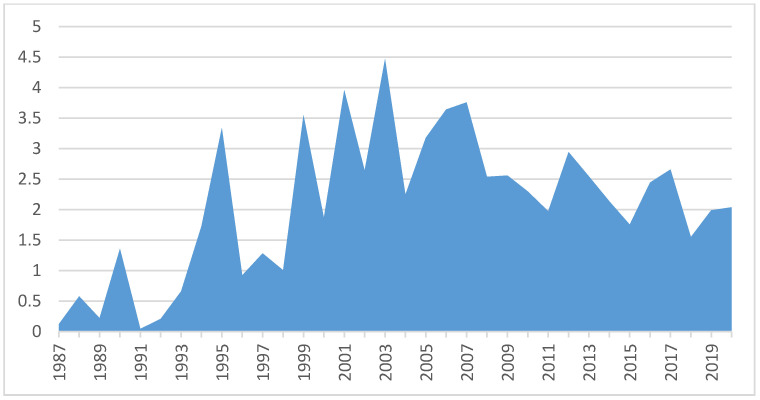
Average of citations per year.

An analysis of citations per year (Figure 2) shows an increasing trend until 2006. Although the trend has been decreasing in the last decade, citations per year have averaged more than two.

**Figure 3:**
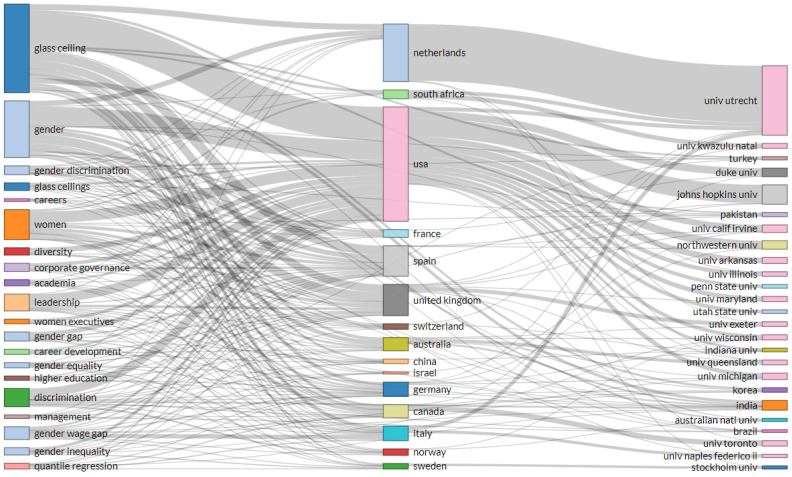
Three-fold analysis of glass ceiling literature.

In addition to studying the scientific production and citations by year, it is worthwhile to explore the main topics, the countries where the articles have been published, and their authors’ affiliations. Figure 3 shows a three-way analysis with keywords on the left, countries in the middle, and affiliations on the right. It can be seen that the United States, in absolute terms, is the country that has published the most on all topics related to the glass ceiling, followed by the Netherlands, which has published mainly on the glass ceiling and gender. Other countries of particular relevance in all topics are Spain and the United Kingdom. The universities with the highest number of publications are Utrecht University, with collaborations with other universities in the United States, South Africa, Italy, and Johns Hopkins University.

### 3.2. Sources

#### 3.2.1. Core Journals

A bibliometric analysis requires a study of the sources where the papers have been published. When analyzing the results, the first thing we found was the large number of sources where the papers on the “glass ceiling” were published. In total, there are 580 WoS sources, which demonstrates the multidisciplinary nature of the subject and the fact that there are not many specialized journals on this topic. There are various fields of knowledge from which the subject of the glass ceiling is studied. An analysis of the journal categories in WoS confirms the multidisciplinarity of the subject. Considering the first of the categories to which the journal belongs, the most frequent category is Psychology, accounting for 18.6% of the journals. In the second place, 15.7% of the journals publishing papers on the glass ceiling belong to the category Economics. Sociology is in third place, with 12.9% of the journals. Women Studies is next, with 7.2% of the journals, and the fifth place goes to Management and Industrial Relations and Labor, with 5.8%. Less frequently, papers have been published in journals from 16 other categories, including Business, Political Science, Engineering, Law, Ethics, Gender Studies, etc. 

Of the total number of publications, seven of them published more than five articles, representing 9.23% of the scientific production. The journal that published the most articles on the subject is *Gender in Management* (26 papers). The second position is *Business Ethics* (13 papers), followed by *Gender & Society* (9 papers) and the *International Journal of Manpower* (8 articles). On the opposite side of the ranking are 465 journals that have only published one paper on the glass ceiling, representing 56.5% of the scientific production. 

One measure of the quality of scientific journals is the Journal Impact Factor (JIF), which was created by Eugene Garfield [35]. The JIF is an indicator used to assess the international status and academic impact of journals [36] and is also a valuable indicator for evaluating the visibility of journals [37]. According to its JIF, each journal’s position will determine the quartile in which it is placed, with Q1 being the most important. The quartiles to which the journals belong have been analyzed, and 44.12% of the journals are, in some of their categories, in the first quartile, which indicates their high quality. The journals with the highest number of articles published on the glass ceiling, *Gender in Management*, is in Q3, the *Journal of Business Ethics* and *Gender & Society* are in Q1, and the *International Journal of Manpower* is in Q3.

Bradford [38] hypothesized that a few journals might publish the most papers on a specialized subject: for any of single discipline, one-third of the resources represent the most frequently journals of that discipline designated as the core source of publication. The practical application of Bradford’s law provides the mechanisms to select the journals that are the most productive and the most relevant to cover a given area of knowledge [39].

This paper observed that Bradford’s core (zone 1) consists of 75 journals that published 280 articles (see Table 2). Zone 1 represents the leading sources for publishing papers on the glass ceiling and represents 13% of the total sources. The average number of articles published per journal in this first group is 3.73 articles. Zone 2 consists of 285 papers published in 236 journals, while the remaining papers belonging to zone 3 were published in 269 different journals. The average number of articles published per journal decreases to 1.21 in the second group and 1 in the third group. Therefore, it is confirmed that the scientific production of the subject under study is uneven, following the guidelines of Bradford’s law. The literature dispersion in the sources is also shown because out of the 580 sources analyzed, only one article on the glass ceiling was published in 80% of them.

Da Rocha Grangeiro et al. [6] indicate that most articles on gender inequality in organizations are published in *Gender in Management* and *Journal of Business Ethics*. Therefore, these two journals are now consolidated as the most relevant for publishing work on the glass ceiling.

#### 3.2.2. Source Dynamics

**Figure 4:**
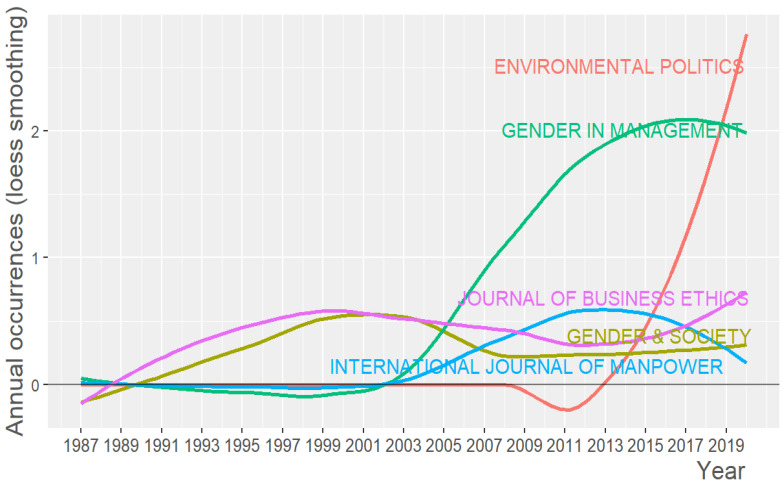
Source growth based on the number of publications per year.

The evolution of the top five publications from 1987 to 2020 is shown in Figure 4. Following Nasir et al. [34], we use the Loess smoothing technique, which is the locally weighted smoothing use regression analysis to demonstrate the smooth line with the help of a time plot or scatter plot [40]. Loess smoothing helps to better understand trends over time [40]. 

The journals analyzed are *Gender in Management*, *Journal of Business Ethics*, *Gender & Society, International Journal of Manpower*, and *Environmental Politics*. Since 2002, there has been an increase in the number of publications in *Gender in Management*, which is the main journal for publishing glass ceiling papers, although the number has decreased in recent years. In 2020, *Environmental Politics* was the journal with the highest value, being the journal with the highest growth since 2013. The *Journal of Business Ethics* has also seen significant growth over the last five years. *Gender & Society* remains the same, and the *International Journal of Manpower* has experienced a decrease in the number of papers published on the subject under study since 2013.

### 3.3. Documents

#### 3.3.1. Most Cited Papers Globally

One of the indications of the quality of academic articles is the number of citations [41]. This study analyzes 823 papers on the glass ceiling, which together have had 18,011 citations in WoS. To determine the most influential papers, we based our analysis on the 10 most cited papers (Table 3).

The most cited paper, which occupies the first position in the ranking, is Ridgeway [42], which has 509 citations since its publication, which means an annual average of 24.24 citations. This work has 2.5% of the total citations of all the documents analyzed. It is a theoretical paper in which, using expectation states theory, Ridgeway [42] describes how gender status beliefs create a network of restrictive expectations and interpersonal reactions that is one of the main causes of the glass ceiling. The paper by Ryan and Haslam [43] ranks second with 421 total citations and exceeds the previous paper in the number of citations per year. They analyze the performance of FTSE 100 companies on the London Stock Exchange before and after the appointment of a male or female board member.

In the third position is the work of Arulampalam et al. [44], with 342 total citations. The authors analyze gender wage gaps by sector in the wage structure in eleven European countries. They found that the gap generally widened toward the top of the wage distribution (the “glass ceiling” effect) and, in some cases, also widened at the bottom (the “sticky floor” effect).

Albrecht et al. [45] ranked fourth with 328 total citations. In their paper, they found that there is a wage gap in Sweden, which is a strong glass ceiling effect.

The paper by Tesch et al. [46] has 324 citations in total and ranks fifth. They conclude that women faculty in US medical schools rise more slowly than men. The gender differences in rank achieved are not explained by productivity or differential attrition in academic medicine [41]. The following paper in the ranking stands out in terms of average citations per year. It ranks sixth in terms of total citations, but it is the article with the highest number of citations per year, namely 30.8. In this paper, Adams and Funk [47] find significant differences between male and female managers’ risk values and attitudes.

The seventh paper in the ranking is published by Lyness and Heilman [48] and has 289 total citations. These authors examined the relationships of gender and job type (i.e., line or staff) with the performance appraisals of 448 senior managers and the relationships of performance appraisals with promotions over the following two years, finding significant differences between men and women. 

The paper by Eagly and Carli [49] ranked eighth and has 285 total citations. In this paper, the authors argue that the glass ceiling is the sum of many obstacles that women face in their working lives, and it is only when all the pieces are put together that a new picture emerges of why women do not make it to the top. 

Reynolds [50] ranks second to last of the top 10 articles. This paper has a total of 278 citations. The paper analyzes the factors that hinder or facilitate women’s access to political representation.

Finally, Maume [51] closes the list of the ten most cited papers with 243 citations. This paper examines race and gender composition in occupation of origin on movement to a managerial position. Maume [51] finds an impact of a “glass escalator” for white men, a “glass ceiling” for others, and contradicts the notion of a “declining importance of race”.

#### 3.3.2. Main Words

Keywords enable readers of the papers to determine the conceptual structure of a discipline without consulting the full text of the paper [52]. A total of 1377 keywords were found in the articles analyzed. The most frequent words used in the literature on the glass ceiling were extracted using Bliblioshiny software and are shown in Table 4. These words have the highest number of occurrences in the keywords of the articles. The most frequently used word in the glass ceiling articles is “women”, which appears in 166 articles, representing 12% of the scientific output. The second most used word is “gender”, representing 10% of the total number of publications. In third place, and at a greater distance, is the word “work”, which is repeated 56 times. Other most frequent words with similar occurrence are “management”, “discrimination”, “female”, “men”, “performance”, “race”, and “sex” appear less than 50 times.

The literature on the glass ceiling relates this research topic mainly to women’s and gender issues. This phenomenon occurs in the work environment, especially in management positions, leading to discrimination between men and women. Therefore, many researchers point to the terms “work”, “management”, “discrimination”, “female”, and “men” as keywords. Research has shown significant differences in performance between men and women who reach management positions [43,51,53,54,55]. The frequent use of the terms “race” and “sex” proves that the glass ceiling phenomenon appears not only for gender but also for other reasons such as race. As mentioned above, [51] explored these issues in more detail.

**Figure 5:**
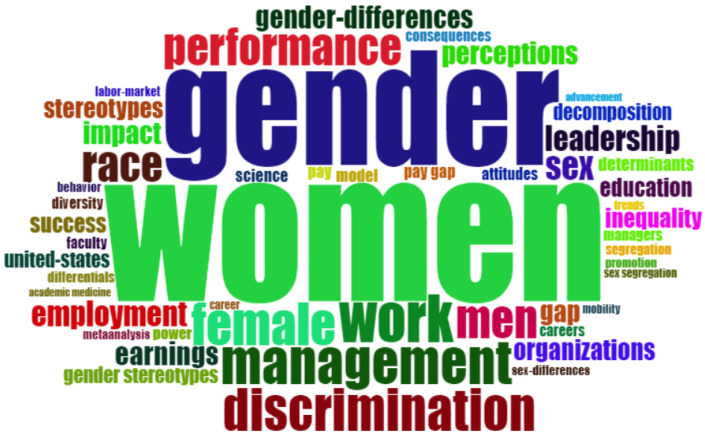
Word cloud.

Figure 5 shows the cloud of the most frequent words, where the more significant the word is, the greater the frequency of use in the papers. As mentioned above, the words women and gender stand out. It should be noted that among the ten most frequent keywords, many terms could be considered synonyms, thus reinforcing the concept they represent. Therefore, the words “gender” and “sex” as well as “women” and “female” are found to be similar. Other studies point to “men” as a keyword closely related to the previous terms.

**Figure 6:**
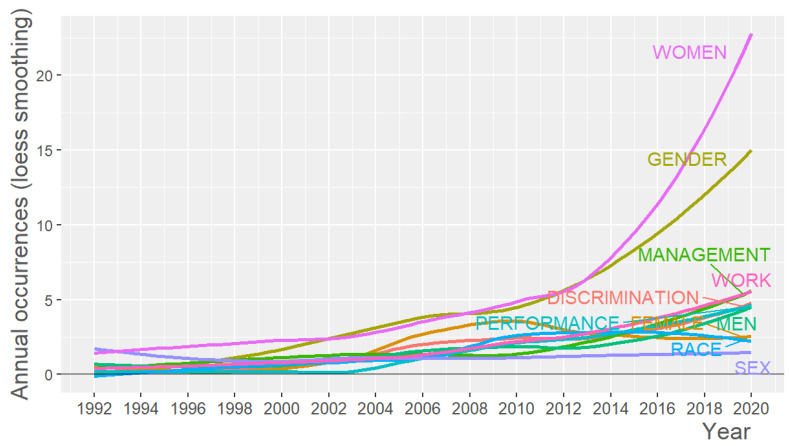
Word growth over time.

The analysis of the keywords is completed by analyzing their evolution over time (Figure 6). The graph was made using the Loess smoothing technique [40]. It can be seen in the graph that the use of the keywords that have been growing most, in parallel until 2013, has been “women” and “gender”. From 2014 onwards, “women” has been used more than “gender,” Its growth and use are much higher in the last three years. The word “female” began to decrease in use from 2009 onwards, with “men” also experiencing a slight decrease between 2010 and 2013. “Race” has experienced a drop in usage from 2015 onwards. The rest of the keywords have followed an upward trajectory.

### 3.4. Authors

#### 3.4.1. Most Relevant Authors

The significance of authors can be measured using several indicators. The total number of citations received for an author’s paper is used to indicate the influence of an author in the academic community. The total citations allow us to identify the most relevant authors in a given field of knowledge [28]. The impact of researchers is measured using the H-Index, by comparing papers with citations [56]. Table 5 shows the indicators for the 22 authors who have received more than 300 citations. 

Based on the total number of citations received, the most relevant authors are Ryan and Haslam, with 682 and 680 citations. This relevance is reinforced by the results of the H-Index, which identifies these authors as the ones with the highest impact, as their H-Index is higher than the rest. Ryan is also the author who has published the highest number of papers on the glass ceiling, six papers. This author received an average of 114 citations per paper, so we cannot say that he was the most influential author for any of his papers. In this respect, it is worth mentioning Ridgeway, who obtained a total of 509 citations with a single paper. The third highest-ranking is Lyness, with 563 citations for his three published papers. Booth has 377 citations for two published papers. However, it is interesting to note that Arulampalam and Bryan have received 342 citations for a single paper.

We complete the analysis of the most relevant authors by applying Lotka’s law to our data. Lotka’s law is a bibliometric law, developed by Alfred Lotka in 1926, on the distribution of authors according to their productivity [57,58]. According to this law, the largest number of authors publish the smallest number of papers. In contrast, the smallest number of authors publish the largest number of papers, this being the most prolific group. The results referring to Lotka’s law for our data are shown in Table 6.

It can be seen that only two authors out of the 1764 authors in the sample have published five or more papers on the glass ceiling, representing 0.20% of the authors. While for 95% of the authors, 1675 of them have published only a single paper on the topic.

#### 3.4.2. Most Relevant Affiliation

Table 7 shows the top 10 countries according to the number of publications and citations they have achieved for published work on the glass ceiling.

The most relevant country in terms of both the number of papers and citations received is the United States, with 546 papers and 10,374 citations. It is followed by the United Kingdom with 133 papers and 1951 citations. Spain is in third place with 79 papers, while in terms of citations received, it is in fourth place with 576 citations. Australia follows this with 1337 citations. Canada holds fourth place with 59 papers. Other countries in the top 10 are Italy, France, Germany, the Netherlands, and China.

Table 8 shows the most relevant affiliations. Only those universities that occupy the first three positions have been included. 

The University of Utrecht published 17 papers on the glass ceiling. In the second place, with nine papers, are the Universities of Duke, Johns Hopkins, and California Irvine. In third place are the Universities of Exeter, Queensland, and Wisconsin, with eight papers each.

### 3.5. Conceptual Structure

In this section, we present the conceptual structure of the research on the glass ceiling. To do so, we first make, based on the relationships between the keywords, a co-occurrence network that will allow us to assess multiple topics on the glass ceiling over time. Second, we construct a two-dimensional matrix where we place the word networks forming the “thematic map”. Finally, we analyze the evolution of this thematic map.

#### 3.5.1. Co-Occurrence Network

Co-word analysis is a method used to gain insight into the meaning of the content of papers and map the structure and development of a scientific discipline [20,59,60]. The co-word analysis method is based on two main premises: first, keywords are carefully selected by authors to represent the content of articles adequately; second, the co-occurrence of two topics in different articles indicates the correlation between them [60].

**Figure 7:**
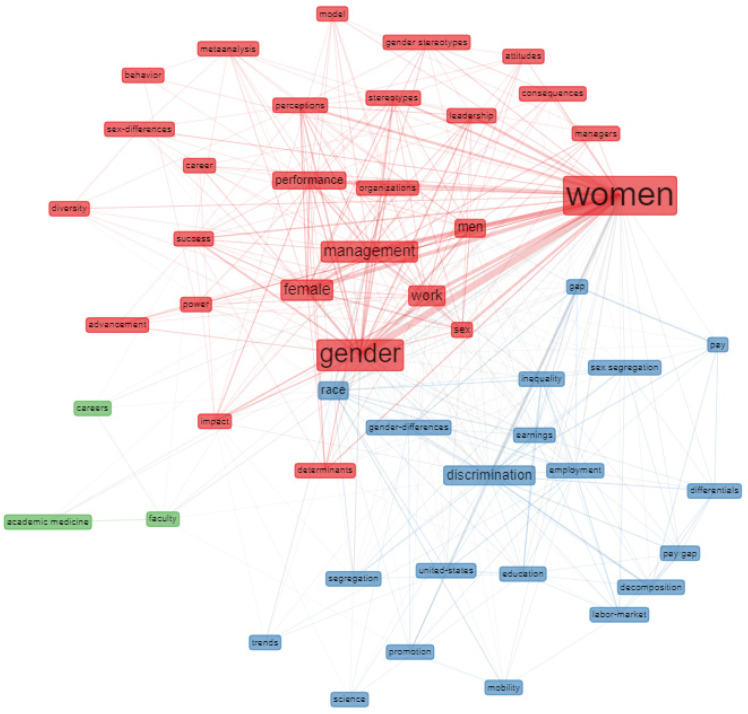
Co-occurrence network.

The co-occurrence network shown in Figure 7 has been generated with the biblioshiny software from the keywords plus. It can be seen that the keyword relationships are distributed in three clusters intertwined with each other through relationships between some keywords. The clusters represent groups of textual information that can be understood as semantic or conceptual groups of different topics of the research field [61]. 

The main cluster is the red cluster, and the word “women” has a dominant position followed by “gender”, “management”, “work”, “female”, “men”, and “performance”. This implies that these keywords have served as important centers in the collaborative word network linking other keywords. The set of related words shows that glass ceiling researchers have focused their work on women’s problems in managerial positions in organizations. The results are consistent with Tekeli [10], who states that the phenomenon “glass ceiling” has been generally used in the literature on women workers. The blue cluster is dominated by “discrimination” and “race” followed by other themes such as “gender-differences”, “gap”, “pay”, “employments”, “earnings”, “education”, “promotion”, etc. This group of related words identifies research that has focused on analyzing the factors or causes that lead to the emergence of the “glass ceiling” phenomenon. Finally, the network of co-occurrences shows a third, smaller, green cluster that represents the studies that have been carried out on the glass ceiling in the academic world.

#### 3.5.2. Thematic Map

**Figure 8:**
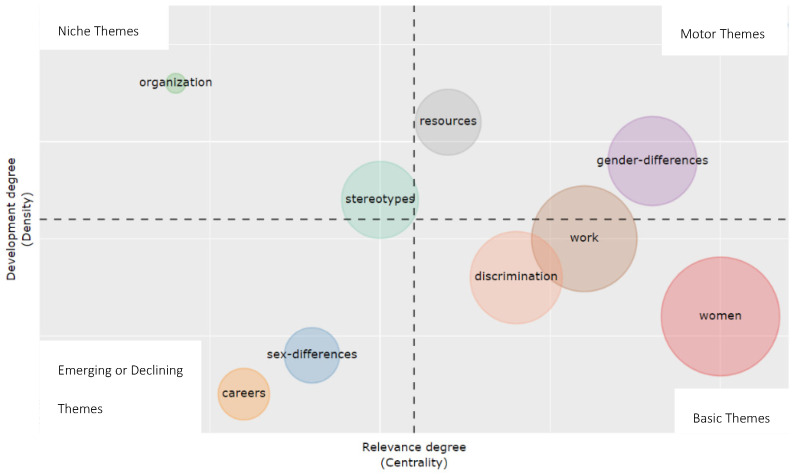
Thematic map.

The research themes identified in the keyword co-occurrence relationship have been represented in a two-dimensional thematic map based on Callon et al. [62] density and centrality scores (Figure 8). The main domain refers to all issues related to women, work, and discrimination, and it is represented on the bottom right, with high centrality and low density. This means that these themes are essential and cross-cutting in all glass ceiling research. With a high level of centrality and density, the domain “gender differences” appears, which means that we can identify this theme as one of the driving themes of glass ceiling research, together with “resources”. The literature shows how the management levels of the organization do not offer the same resources to all people, with certain minorities facing barriers to occupying certain positions [63]. In quadrant four of the graph, the domain “stereotypes” is shown. This indicates that this theme is considered peripheral and with great potential of development. With a low level of both centrality and density, we identify the domains “careers” and “sex-differences.” These could be interpreted as emerging themes within glass ceiling research. Currently, more research is being done on the obstacles for certain minorities to make a career within the organization at all levels, not only the study of the glass ceiling at the managerial level. 

The traditional problems of glass ceilings or barriers in the top management of companies are extending to all levels of the organization, and they are not exclusive to the highest positions in the organizational structure. In this sense, the literature has traditionally dealt with gender discrimination focused on management levels. However, we note that gender discrimination at other levels has not had the same development in other research, making gender discrimination in positions other than top management an emerging issue that needs further development. The structure of glass ceiling research is complex and rich with a high degree of specialization.

#### 3.5.3. Thematic Evolution

**Figure 9:**
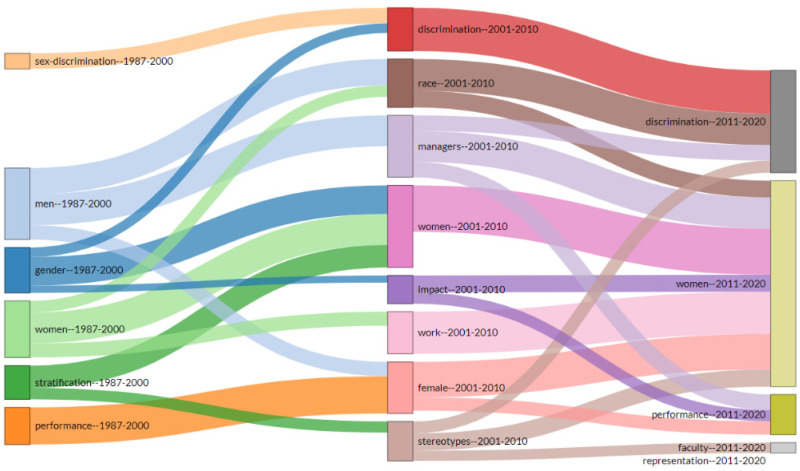
Thematic evolution.

In addition to the thematic map, there is the thematic evolution (Figure 9), which shows the historical development of the glass ceiling literature. This thematic evolution is analyzed based on the keyword plus. As can be seen, the thematic evolution can be divided into three main time segments. The first segment is from 1987 to 2000, the second is from 2001 to 2010, and the last is from 2011 to 2020. The topics have evolved over time. According to our results, in the first period, the literature on the glass ceiling is widely spread over several topics. However, the papers deal primarily with issues such as men, women, gender, and to a lesser extent, performance stratification and sex discrimination. In the second period, from 2001 to 2010, the most frequent themes are women and managers, followed by race and discrimination. This indicates that in this decade, the studies focus on the problems that lead to the emergence of the glass ceiling at the managerial level. Finally, between 2010 and 2020, the themes increasingly evolve toward women, discrimination, and performance. 

## 4. Conclusions

There are still barriers in organizations that prevent certain groups, especially women, from continuing their professional careers and accessing management positions. Researchers have been analyzing the “glass ceiling” phenomenon for years, but it is necessary to know what has been published on the subject to offer new needs and lines of research. Therefore, this paper has studied the relevance of the literature on the glass ceiling by analyzing the fundamental aspects, and the topics analyzed in the literature and their relationships (conceptual structure). 

The absence of studies conducting a descriptive review of the literature on the “glass ceiling”, including its evolution, authors, main affiliations, sources, and countries, as well as its conceptual structure, prompted us to develop this bibliometric study of articles published in WoS in the period 1987–2020. An essential contribution of this work is the temporal breadth over which the study was conducted, as there has been no other research that has previously analyzed papers on the glass ceiling over such a long period in the leading academic database. This work complements previous studies that have explored the literature on gender inequalities in organizations [6]. Firstly, a descriptive bibliometric analysis was developed, and, secondly, the subtopics analyzed in the literature and their relationships (conceptual structure) were identified through a co-word analysis, which is one of the least common bibliometric techniques in business studies (13.6%) [20]. 

We identified some relevant findings from the descriptive bibliometric study. First, the glass ceiling phenomenon has been a growing research topic and is nowadays a hot topic in business management research and gender literature. Second, the broad spectrum of journals where the analyzed articles are published shows the multidisciplinarity of the topic. The journals *Gender in Management* and *Journal of Business Ethics* are the ones with the highest number of articles published. These two journals are also the most relevant publications on inequalities in the workplace [6]. In recent years, the journal *Environmental Politics* has experienced the greatest growth in publications on the glass ceiling. However, the glass ceiling to which most of the articles published in this journal refer are those related to the barriers to achieving environmental status. Third, concerning authors, the most relevant in terms of the number of citations and their H-Index are Ryan and Haslam, who are both from the University of Exeter. The most cited paper is Ridgeway [42], from Stanford University. Fourth, the most relevant countries in terms of publications on the subject are the United States and the United Kingdom. The most productive universities in terms of the number of papers published are Utrecht University (Netherland), Duke University, Johns Hopkins University, and the University of California, Irvine (USA).

With regard to the conceptual structure, a co-word analysis was carried out, from which the following conclusions were drawn. First, the words “women” and “gender” are the most used keywords and the ones that have experienced the greatest growth in recent years. Second, the co-occurrence network of co-words has allowed us to identify the main themes of analysis in terms of the “glass ceiling”. A dominant theme in the published studies is the problems faced by women in managerial positions in organizations. The second topic of study is factors or causes leading to the glass ceiling phenomenon, identifying multiple grounds for discrimination. Thirdly, the research structure has proved to be complex and rich, with a high degree of specialization. The theme of women is consolidated as a basic theme within glass ceiling research, and aspects related to gender difference and resources in the workplace are identified as a driving theme.

### 4.1. Practical Implications

This paper makes important practical contributions. It has critically assessed the literature on the glass ceiling by identifying the most relevant journals for researchers to publish their work on the topic, leading authors who are studying the topic, the most cited papers, and the most common research topics. Our analysis allows us to identify new research trends that academics and researchers can take advantage of in their future work. Academic institutions interested in studying this topic have a guide to the leading journals and can subscribe to those not yet available to their researchers.

### 4.2. Limitations and Future Research Agenda

The study’s main limitations stem from the selection of the sample of articles and the bibliometric techniques used. We used a single database (WoS) to select the documents under study. However, other databases such as Scopus or Abi-inform could also be used to increase the number of documents analyzed. Concerning bibliometric techniques, a co-citation analysis or bibliographic coupling could be carried out.

As future lines of research, this glass ceiling review should be complemented with other bibliometric techniques to understand the research topic’s social and intellectual structure. Similarly, data searches could be extended to new search queries and other terms to search for the resulting articles. Another limitation may be related to the fact that the study used abstracts and titles rather than full text to select papers. However, as already pointed out by Vázquez-Carrasco and López-Pérez [64] and Lechuga Sancho et al. [25], although it is necessary to mention certain research limitations, we should also bear in mind that the shortcomings of these studies are inherent to bibliometric analysis as a genre.

Based on the most important issues identified and considering that the glass ceiling is a hot topic in business management, more research is still needed on the problems faced by groups other than women. Little is known about the glass ceiling in terms of ethnicity or sexual orientation. It would be interesting to learn about the problems faced by people of color and LGBTI people in gaining access to management positions. Similarly, the “glass ceiling” issue in specific economic sectors remains unexplored; for example, it is interesting to learn about this phenomenon of inequality in the tourism sector, which is a sector with predominantly female employment.

## Figures and Tables

**Table 1:** Descriptive characteristics of the literature on the “glass ceiling”.

Description	Results
Documents	823
Sources	580
Keywords plus (ID)	1377
Author’s keywords (DE)	1622
Timespan	1987:2020
Average citations per documents	21.88
Authors	1764
Author appearances	1875
Authors of single-authored documents	294
Authors of multi-authored documents	1470
Single-authored documents	308
Documents per author	0.467
Authors per document	2.14
Co-Authors per document	2.28
Collaboration index	2.85

**Table 2:** Journals classification according to Bradford’s law.

SO	Rank	Freq	cumFreq	Zone
Gender in management	1	26	26	Zone 1
Journal of business ethics	2	13	39	Zone 1
Gender & society	3	9	48	Zone 1
International journal of manpower	4	8	56	Zone 1
Environmental politics	5	7	63	Zone 1
Gender work and organization	6	7	70	Zone 1
Equality diversity and inclusion	7	6	76	Zone 1
Applied economics	8	5	81	Zone 1
Applied economics letters	9	5	86	Zone 1
Behavior analyst	10	5	91	Zone 1
Public administration review	11	5	96	Zone 1
Sex roles	12	5	101	Zone 1
American sociological review	13	4	105	Zone 1
Behavior therapy	14	4	109	Zone 1
Human resource management	15	4	113	Zone 1
International review of applied economics	16	4	117	Zone 1
Journal of applied psychology	17	4	121	Zone 1
Journal of economic behavior & organization	18	4	125	Zone 1
Journal of economic inequality	19	4	129	Zone 1
Leadership quarterly	20	4	133	Zone 1
Women & politics	21	4	137	Zone 1
Academy of management journal	22	3	140	Zone 1
Administrative sciences	23	3	143	Zone 1
Advances in developing human resources	24	3	146	Zone 1
Affilia-journal of women and social work	25	3	149	Zone 1
Agenda-empowering women for gender equity	26	3	152	Zone 1
American economic review	27	3	155	Zone 1
Asian women	28	3	158	Zone 1
British journal of management	29	3	161	Zone 1
Feminist economics	30	3	164	Zone 1
Feminist theology	31	3	167	Zone 1
Frontiers in psychology	32	3	170	Zone 1
Gender and education	33	3	173	Zone 1
Global business review	34	3	176	Zone 1
Hastings law journal	35	3	179	Zone 1
Human resources for health	36	3	182	Zone 1
Industrial relations	37	3	185	Zone 1
International journal of hospitality management	38	3	188	Zone 1
International journal of Indian culture and business management	39	3	191	Zone 1
Jama-journal of the American medical association	40	3	194	Zone 1
Journal of business research	41	3	197	Zone 1
Journal of career development	42	3	200	Zone 1
Journal of labor research	43	3	203	Zone 1
Journal of management	44	3	206	Zone 1
Journal of vocational behavior	45	3	209	Zone 1
Journal of women politics & policy	46	3	212	Zone 1
Labour economics	47	3	215	Zone 1
Public relations review	48	3	218	Zone 1
Scientist	49	3	221	Zone 1
Social science research	50	3	224	Zone 1
Strategic management journal	51	3	227	Zone 1
Sustainability	52	3	230	Zone 1
Transylvanian review of administrative sciences	53	3	233	Zone 1
Wisconsin law review	54	3	236	Zone 1
Work and occupations	55	3	239	Zone 1
Work employment and society	56	3	242	Zone 1
American journal of surgery	57	2	244	Zone 1
American psychologist	58	2	246	Zone 1
Annals of the American academy of political and social science	59	2	248	Zone 1
Asian American journal of psychology	60	2	250	Zone 1
Asian journal of womens studies	61	2	252	Zone 1
British journal of social psychology	62	2	254	Zone 1
British journal of sociology	63	2	256	Zone 1
Cambio-rivista sulle trasformazioni sociali	64	2	258	Zone 1
Canadian public policy-analyse de politiques	65	2	260	Zone 1
Chemical & engineering news	66	2	262	Zone 1
China economic review	67	2	264	Zone 1
Cornell hospitality quarterly	68	2	266	Zone 1
Economics bulletin	69	2	268	Zone 1
Engineering construction and architectural management	70	2	270	Zone 1
Ethnic and racial studies	71	2	272	Zone 1
European management journal	72	2	274	Zone 1
European political science	73	2	276	Zone 1
Evidence-based HRM-a global forum for empirical scholarship	74	2	278	Zone 1
Gender, careers and inequalities in medicine and medical education: international perspectives	75	2	280	Zone 1

**Table 3:** Top 10 most cited papers.

Ranking	Title	Authors	Year	Total Citations	TC Per Year
1	Gender, status, and leadership.	Ridgeway, C.L.	2001	509	24.2381
2	The glass cliff: Evidence that women are over-represented in precarious leadership positions.	Ryan, M.K., and Haslam, S.A.	2005	421	24.7647
3	Is there a glass ceiling over Europe? Exploring the gender pay gap across the wage distribution.	Arulampalam, W., Booth, A.L., and Bryan, M.L.	2007	342	22.8
4	Is there a glass ceiling in Sweden?	Albrecht, J., Björklund, A., and Vroman, S.	2003	328	17.2632
5	Promotion of women physicians in academic medicine: glass ceiling or sticky floor?	Tesch, B.J., Wood, H.M., Helwig, A.L., and Nattinger, A.B.	1995	324	12
6	Beyond the glass ceiling: Does gender matter?	Adams, R.B., and Funk, P.	308	30.8	
7	When fit is fundamental: performance evaluations and promotions of upper-level female and male managers.	Lyness, K.S., and Heilman, M.E.	289	18,0625	
8	Women and the labyrinth of leadership.	Eagly, A.H. and Carli, L.L.	2007	285	19
9	Women in the legislatures and executives of the world: Knocking at the highest glass ceiling.	Reynolds, A.	1999	278	12.087
10	Glass ceilings and glass escalators: Occupational segregation and race and sex differences in managerial promotions.	Maume, Jr., D.J.	1999	243	10.5652

**Table 4:** Most frequent words.

Words	Occurrences
Women	166
Gender	134
Work	56
Management	53
Discrimination	51
Female	50
Men	45
Performance	44
Race	43
Sex	34

**Table 5:** Most relevant authors.

Author	TC	NP	TC/NP	h_Index
Ryan MK	682	6	114	4
Haslam SA	680	5	136	4
Lyness KS	563	3	188	3
Ridgeway CL	509	1	509	1
Booth AL	377	2	189	2
Albrecht J	345	2	173	2
Vroman S	345	2	173	2
Arulampalam W	342	1	342	1
Bryan Ml	342	1	342	1
Bjorklund A	328	1	328	1
Carli LL	325	2	163	2
Eagly AH	325	2	163	2
Baxter J	324	3	108	3
Wright EO	324	3	108	3
Helwig Al	324	1	324	1
Nattinger Ab	324	1	324	1
Tesch Bj	324	1	324	1
Wood HM	324	1	324	1
Cook A	313	3	104	3
Glass C	313	3	104	3
Adams Rb	308	1	308	1
Funk P	308	1	308	1

**Table 6:** Lotka’s law.

Documents Written	N. of Authors	Proportion of Authors
1	1675	95.00%
2	72	4.10%
3	15	0.90%
5	1	0.10%
6	1	0.10%

**Table 7:** Top 10 countries by publications and citations.

Country	Freq	Country	Total Citations
USA	546	USA	10,374
UK	133	UNITED KINGDOM	1951
Spain	79	AUSTRALIA	1337
Canada	59	SPAIN	576
Australia	58	FRANCE	426
Italy	47	CANADA	415
France	44	NETHERLANDS	319
Germany	43	GERMANY	247
Netherlands	42	ITALY	233
China	32	CHINA	230

**Table 8:** Most relevant affiliations.

Rank	Affiliations	Articles
1	Univ Utrecht	17
2	Duke Univ	9
	Johns Hopkins Univ	9
	Univ Calif Irvine	9
3	Univ Exeter	8
	Univ Queensland	8
	Univ Wisconsin	8

## Data Availability

Data is available in the Web of Science database: https://www-webofscience-com.bibezproxy.uca.es/wos/alldb/basic-search (accessed on 26 July 2021).

## References

[1] Carvalho I., Costa C., Lykke N., Torres A. (2019). Beyond the glass ceiling: Gendering tourism management. Ann. Tour. Res..

[2] Ramos A. (2005). Mujeres directivas: Un valor en alza para las organizaciones laborales. Cuad. Geogr..

[3] Parcheta N., Kaifi B.A., Khanfar N.M. (2013). Gender inequality in the workforce: A human resource management quandary. J. Bus. Stud. Q..

[4] Purcell D. (2013). Baseball, beer, and bulgari: Examining cultural capital and gender inequality in a retail fashion corporation. J. Contemp. Ethnogr..

[5] Jauhar J., Lau V. (2018). The ‘glass ceiling’ and women’s career advancement to top management: The moderating effect of social support. Glob. Bus. Manag. Res..

[6] Da Rocha G.R., Silva L.E.N., Esnard C. (2021). I broke the glass ceiling, now what? Overview of metaphors to explain gender inequality in organizations. Int. J. Organ. Anal..

[7] Cotter D.A., Hermsen J.M., Ovadia S., Vanneman R. (2001). The glass ceiling effect. Soc. Forces.

[8] Wirth L. Women in management: Closer to breaking through the glass ceiling. In Women, Gender and Work. https://www.ilo.org/public/libdoc/ilo/2001/101B09_102_engl.pdf.

[9] Huete R., Brotons M., Sigüenza M.C. (2016). La desigualdad entre mujeres y hombres en el sector hostelero español. Estud. y Perspect. en Tur..

[10] Tekeli H.N. (2019). Women’s employment in tourism sector in turkey, issues faced, and the effect of glass ceiling syndrome on women workforce in tourism. Soc. Sci..

[11] Bendl R., Schmidt A. (2010). From ‘Glass Ceilings’ to ‘Firewalls’—different metaphors for describing discrimination. Gend. Work. Organ..

[12] Sánchez-Teba E.M., Benítez-Márquez M.D., Porras-Alcalá P. (2020). Gender Diversity in Boards of Directors: A Bibliometric Mapping. J. Open Innov. Technol. Mark. Complex..

[13] Jurkus A.F., Park J.C., Woodard L.S. (2011). Women in top management and agency costs. J. Bus. Res..

[14] Adams R.B., Ferreira D. (2009). Women in the boardroom and their impact on governance and performance. J. Financ. Econ..

[15] Berezinets I.V., Garanina T.A., Ilina Y.B. (2018). Social capital of women directors and financial performance of a company: Empirical study. Russ. Manage. J..

[16] Usman M., Farooq M.U., Zhang J., Makki M.A.M., Khan M.K. (2019). Female directors and the cost of debt: Does gender diversity in the boardroom matter to lenders?. Manag. Audit. J..

[17] Setó-Pamies D. (2015). The relationship between women directors and corporate social responsibility. Corp. Soc. Responsib. Environ. Manag..

[18] Rodríguez-Fernández M., Gaspar-González A.I., Sánchez-Teba E.M. (2020). Does diversity in top management teams contribute to organizational performance? The response of the IBEX 35 companies. Soc. Sci..

[19] Ruiz-Jiménez J.M., Fuentes-Fuentes M.M., Ruiz-Arroyo M. (2016). Combination capability and innovation: The effects of gender diversity on top management teams in technology-based firms. J. Bus. Ethics.

[20] Zupic I., Čater T. (2015). Bibliometric methods in management and organization. Organ. Res. Methods.

[21] Da Rocha G.R., Rodrigues M.S., Silva L.E.N., Esnard C. (2019). Scientific Metaphors and Female Representativeness in Leadership Positions: A Bibliometric Analysis. Rev. Psicol. Organ. Trab..

[22] Corsi M., D’Ippoliti C., Zacchia G. (2019). On the evolution of the glass ceiling in italian academia: The case of economics. Sci. Context.

[23] Ngaage L.M., Ngadimin C., Harris C., Rawes C., Wu Y., Landford W., Rasko Y.M. (2020). The Glass Ceiling in Plastic Surgery: A Propensity-Matched Analysis of the Gender Gap in Career Advancement. Plast. Reconstr. Surg..

[24] Carpenter K., Scullen T., Mathkour M., Dumont A.S., Biro E., Kahn L. (2021). Social Perception and Academic Trends on Women in the Field of Neurosurgery: A Bibliometric Analysis. World Neurosurg..

[25] Lechuga Sancho M.P., Martín-Navarro A., Ramos-Rodríguez A.R. (2020). Information Systems Management Tools: An Application of Bibliometrics to CSR in the Tourism Sector. Sustainability.

[26] De Bakker F.G., Groenewegen P., Den Hond F. (2005). A bibliometric analysis of 30 years of research and theory on corporate social responsibility and corporate social performance. Bus. Soc..

[27] Duque Oliva E.J., Cervera Taulet A., Rodríguez Romero C. (2006). A bibliometric analysis of models measuring the concept of perceived quality in providing internet service. Innovar.

[28] Garfield E. (1979). Is citation analysis a legitimate evaluation tool?. Scientometrics.

[29] Kraus S., Filser M., Eggers F., Hills G., Hultman C. (2012). The entrepreneurial marketing domain: A citation and co-citation analysis. J. Res. Mark. Entrep..

[30] Benavides-Velasco C.A., Quintana-García C., Guzmán-Parra V.F. (2013). Trends in family business research. Small Bus. Econ..

[31] Köseoglu M.A., Okumus F., Putra E.D., Yildiz M., Dogan I.C. (2019). Conceptual structure of lodging-context studies: 1990–2016. J. Hosp. Tour. Res..

[32] Aria M., Cuccurullo C. (2017). Bibliometrix: An R-tool for comprehensive science mapping analysis. J. Informetr..

[33] Janik A., Ryszko A., Szafraniec M. (2020). Scientific Landscape of Smart and Sustainable Cities Literature: A Bibliometric Analysis. Sustainability.

[34] Nasir A., Shaukat K., Hameed I.A., Luo S., Mahboob T., Iqbal F. (2020). A Bibliometric Analysis of Corona Pandemic in Social Sciences: A Review of Influential Aspects and Conceptual Structure. IEEE Access.

[35] Garfield E. (1972). Citation analysis as a tool in journal evaluation. Science.

[36] Campanario J.M. (2011). Large increases and decreases in journal impact factors in only one year: The effect of journal self-citations. J. Am. Soc. Inf. Sci. Technol..

[37] Slim K., Dupré A., Le Roy B. (2017). Impact factor: An assessment tool for journals or for scientists?. Anaesth. Crit. Care Pain Med..

[38] Bradford S.C. (1934). Sources of information on specific subjects. Engineering.

[39] Urbizagástegui Alvarado R. (2016). El crecimiento de la literatura sobre la ley de Bradford. Investig. Bibl..

[40] Royston P. (1992). Lowess Smoothing.

[41] Caon M., Trapp J., Baldock C. (2020). Citations are a good way to determine the quality of research. Phys. Eng. Sci. Med..

[42] Ridgeway C.L. (2001). Gender, status, and leadership. J. Soc. Issues.

[43] Ryan M.K., Haslam S.A. (2005). The glass cliff: Evidence that women are over-represented in precarious leadership positions. Br. J. Manag..

[44] Arulampalam W., Booth A.L., Bryan M.L. (2007). Is there a glass ceiling over Europe? Exploring the gender pay gap across the wage distribution. ILR Rev..

[45] Albrecht J., Björklund A., Vroman S. (2003). Is there a glass ceiling in Sweden?. J. Labor Econ..

[46] Tesch B.J., Wood H.M., Helwig A.L., Nattinger A.B. (1995). Promotion of women physicians in academic medicine: Glass ceiling or sticky floor?. JAMA.

[47] Adams R.B., Funk P. (2012). Beyond the glass ceiling: Does gender matter?. Manag. Sci..

[48] Lyness K.S., Heilman M.E. (2006). When fit is fundamental: Performance evaluations and promotions of upper-level female and male managers. J. Appl. Psychol..

[49] Eagly A.H., Carli L.L. (2007). Women and the labyrinth of leadership. Harv. Bus. Rev..

[50] Reynolds A. (1999). Women in the legislatures and executives of the world: Knocking at the highest glass ceiling. World Politics.

[51] Maume Jr D.J. (1999). Glass ceilings and glass escalators: Occupational segregation and race and sex differences in managerial promotions. Work. Occup..

[52] Romo-Fernández L.M., Guerrero-Bote V.P., Moya-Anegón F. (2013). Co-word based thematic analysis of renewable energy (1990–2010). Scientometrics.

[53] Marlow S., McAdam M. (2013). Gender and entrepreneurship. Int. J. Entrep. Behav. Res..

[54] Robb A.M., Watson J. (2012). Gender differences in firm performance: Evidence from new ventures in the United States. J. Bus. Ventur..

[55] Rosa J.M., Sylla D. (2018). A comparison of the performance of majority female-owned and majority male-owned small and medium-sized enterprises. Int. J. Entrep. Small Bus..

[56] Hirsch J.E. (2005). An index to quantify an individual’s scientific research output. Proc. Natl. Acad. Sci. USA.

[57] Lotka A.J. (1926). The frequency distribution of scientifc productivity. J. Wash. Acad. Sci..

[58] Price D.J.S. (1973). Hacia Una Ciencia de la Ciencia.

[59] Callon M., Courtial J.-P., Turner W.A., Bauin S. (1983). From translations to problematic networks: An introduction to co-word analysis. Soc. Sci. Inf..

[60] Feng J., Zhang Y.Q., Zhang H. (2017). Improving the co-word analysis method based on semantic distance. Scientometrics.

[61] Cobo M.J., López-Herrera A.G., Herrera-Viedma E., Herrera F. (2011). An approach for detecting, quantifying, and visualizing the evolution of a research feld: A practical application to the Fuzzy Sets Theory feld. J. Informetr..

[62] Callon M., Courtial J.P., Penan H. (1995). Cienciometría. La Medición de la Actividad Científica: De la Bibliometría a la Vigilancia Tecnológica.

[63] Carter S., Mwaura S., Ram M., Trehan K., Jones T. (2015). Barriers to ethnic minority and women’s enterprise: Existing evidence, policy tensions and unsettled questions. Int. Small Bus. J..

[64] Vázquez-Carrasco R., López-Pérez M.E. (2013). Small & medium-sized enterprises and Corporate Social Responsibility: A systematic review of the literature. Qual. Quant..

